# Effect of freeze–thaw cycle on physical and mechanical properties and damage characteristics of sandstone

**DOI:** 10.1038/s41598-021-91842-8

**Published:** 2021-06-10

**Authors:** Longxiao Chen, Kesheng Li, Guilei Song, Deng Zhang, Chuanxiao Liu

**Affiliations:** College of Water Conservancy and Civil Engineering, Shandong Agricultural University, Tai’an, 271018 Shandong China

**Keywords:** Civil engineering, Engineering

## Abstract

Rock deterioration under freeze–thaw cycles is a concern for in-service tunnel in cold regions. Previous studies focused on the change of rock mechanical properties under unidirectional stress, but the natural rock mass is under three dimensional stresses. This paper investigates influences of the number of freeze–thaw cycle on sandstone under low confining pressure. Twelve sandstone samples were tested subjected to triaxial compression. Additionally, the damage characteristics of sandstone internal microstructure were obtained by using acoustic emission (AE) and mercury intrusion porosimetry. Results indicated that the mechanical properties of sandstone were significantly reduced by freeze–thaw effect. Sandstone’ peak strength and elastic modulus were 7.28–37.96% and 6.38–40.87% less than for the control, respectively. The proportion of super-large pore and large pore in sandstone increased by 19.53–81.19%. We attributed the reduced sandstone’ mechanical properties to the degenerated sandstone microstructure, which, in turn, was associated with increased sandstone macropores. The macroscopic failure pattern of sandstone changed from splitting failure to shear failure with an increasing of freeze–thaw cycles. Moreover, the activity of AE signal increased at each stage, and the cumulative ringing count also showed upward trend with the increase of freeze–thaw number.

## Introduction

Freeze–thaw action is an important factor affecting rock mechanical properties and has an important influence on engineering stability in cold region^1^. Deterioration of rock properties due to freeze–thaw has recently attracted the attention of many researchers and engineers. Some high-altitude areas and northern areas in China belong to perennial and seasonal cold areas. It is necessary to consider freeze–thaw damage of rocks in such environmental conditions to carry out engineering^2^. When the temperature is low, the pore water freezes into ice, and the volume expansion produces frost heaving force. The development of ice exerts multidirectional tensile effect on rock, expands existing cracks, and makes pore expansion and penetration inside rock produce new macro-cracks^3^. After the temperature rises, the ice melts into water and re-immerses the pore space of rock, which makes the permeability of rock increase^4^. After several freeze–thaw cycles, irreversible freeze–thaw damage occurs and rock strength gradually decreases, which poses a great threat to the stability of geotechnical engineering in cold region^5^.

As is known to all, the freeze–thaw cycles have significant effects on the physical and mechanical parameters of rocks. Previous studies have shown that the uniaxial compressive strength and elastic modulus of rocks decrease exponentially with the increase of freeze–thaw cycles, but the Poisson ratio of rocks increases^6–8^. Moreover, many research investigations generally indicate that increasing the number of freeze–thaw cycles decreases the uniaxial compressive strength, tensile strength, dry density and P-wave velocity of rocks, while the water absorption and porosity of rocks increase^9–11^. Seyed et al. and Yu et al. conducted triaxial compression tests on frozen-thawed rocks and found that the cohesion and internal friction angle of rocks decreased exponentially with the increase of the number of freeze–thaw cycles^4,12^. They found that the correlation between rock compressive strength, confining pressure and cycles can be described by the Mohr–Coulomb strength criterion^13^. In addition, some scholars have found that the water content of specimens has a great influence on the degree of damage caused by freeze–thaw cycles. Through freeze–thaw cycle test on specimens with different water content, Liu et al. and Chen et al. found that the freeze–thaw damage of specimens gradually increased with the increase of water content, and the critical saturation was about 70%^14,15^. Previous studies on freeze–thaw rocks have been carried out in uniaxial compression tests and triaxial compression tests under high confining pressures. However, rocks at tunnel portals that are susceptible to freeze–thaw damage usually have low confining pressures.

There is a certain connection between macro-mechanical properties of rock and its internal micro-characteristics. The process of deterioration and destruction of rock is actually the process of pore structure change in rock^16–18^. In order to obtain the damage mechanism of frozen-thawed rocks, scholars have carried out a lot of detection and analysis on the internal structure characteristics of rocks using modern micro-detection technology. They have carried out nuclear magnetic resonance (NMR) experiments on sandstone after freeze–thaw cycles and found that porosity gradually increases with the increase of cycles^19–21^. Yang et al. and Tim et al. obtained that the damage factor of rock increases linearly with the increasing number of freeze–thaw cycles using CT scanning technique^22,23^. Additionally, the pore structure of rock under freeze–thaw can be observed using scanning electron microscopy (SEM). The existing research generally indicates that the pores of the rock expand and the particles fall off with the increase of freeze–thaw number^2,12,24–26^. However, due to the extremely complex pore structure characteristics of rock, there is little research on quantitative analysis of microstructure changes after rock freeze–thaw damage.

In recent years, a large number of scholars have applied acoustic emission (AE) to the field of rock mechanics for analysis, and have obtained a series of research results by studying the law of crack development in rock by AE signal. Dai et al., Kong et al. and Khazaei et al. identified the growth of rock damage and the onset of structural instability by monitoring AE and described the development of damage using the probability density function of AE events^27–29^. Previous studies have shown that the AE signal of rock can continuously and real-time reflect the dynamic damage evolution process inside the rock. Thus, it has certain theoretical basis to study the freeze–thaw damage process of rock through the change of AE signal.

This study simulates the actual situation of surrounding rock at the tunnel portal which is prone to freeze–thaw damage. The rock samples under different freeze–thaw cycles (0, 7, 14, 21 respectively) were tested for quality and their triaxial compression strength (TCS) was tested to determine the changes of rock physical and mechanical parameters under freeze–thaw cycles. Based on MIP, the variation rule of pore structure in rock was quantitatively analyzed, and damage evolution rule of rock was predicted by combining AE signal. The paper aims to provide advice and information to upgrade and preserve of the existing tunnels in cold regions.

## Materials and methods

### Samples preparation

**Figure 1:**
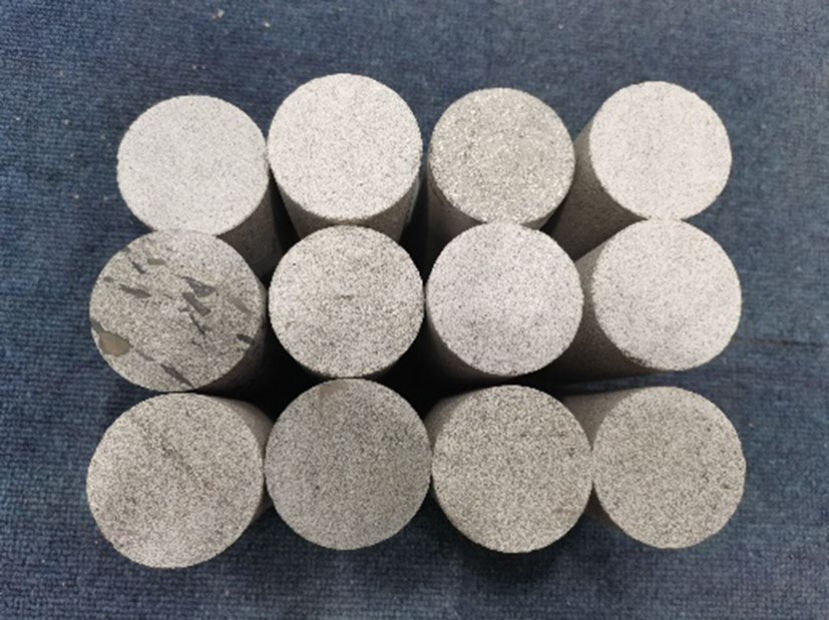
Part of sandstone specimens.

The sandstone used in this study was taken from an in-service tunnel in cold region of northern China. Complete sandstone pillars were drilled at the project site, sealed and transported back to the laboratory to avoid any damage to the sandstone. The sandstone was processed into cylindrical international standard specimens of size 50 mm × 100 mm by drilling, cutting and leveling. The ends of each specimen were parallel to each other. Some of the specimens processed are shown in Fig. 1. In order to avoid the influence of rock dispersion on the test results, specimens with excessive apparent differences or obvious joint failure were removed.

### Experimental design

The main research direction of this test is the difference of physical and mechanical properties, AE signal and microstructure of saturated sandstone samples after different freeze–thaw times. The process of laboratory experiments is explained as follows. First, triaxial compression tests were carried out on the above sandstone and AE signals were recorded during the tests. Secondly, the variation rule of internal microstructure and pore distribution of frozen-thawed sandstone was obtained by MIP. Finally, the physical and mechanical properties, microstructure and development rule of AE signal of sandstone were comprehensively analyzed.

Twelve sandstone specimens with similar physical indices from the same large sandstone were selected and numbered and evenly divided into 4 groups (A, B, C, and D), each with 3 sandstone specimens. All specimens were dried in an oven at 105 ℃ for 48 h and then cooled to room temperature to record the quality of each sample. The method of soaking the specimen in stages was used to ensure that the inside of the specimen is evenly filled with water. The specimens were put into a water tank, first filled with water to 1/4 of the height of the specimens, then filled with water to 1/2 and 3/4 of the height of the specimens every 2 h, and then immersed all the specimens after 6 h. Finally, the specimens absorbed water freely for 48 h and recorded the quality after being saturated with water. General physical parameters of sandstone samples are shown in Table 1.

**Table 1:** Initial physical parameters of sandstone.

Dry density $\rho_{\alpha } /(g \cdot cm^{ - 3} )$	Saturated density $\rho_{s} /(g \cdot cm^{ - 3} )$	Saturated water content $W_{0} /\%$	Porosity $/\%$
2.43	2.49	1.80	7.92

The freeze–thaw cycles of group A, B, C and D were tested, and the cycles were set at 0, 7, 14 and 21 times respectively. Referring to the actual engineering environment where the samples were collected, the freeze–thaw cycle test was set as follows: The saturated specimens were put into the freezing tank at –20 ℃ for 12 h, and then immersed in constant temperature water at + 20 ℃ for 12 h. Each freeze–thaw cycle lasted 24 h. The quality of specimens after freeze–thaw cycle was weighed and the quality change of frozen-thawed sandstone was obtained.

**Figure 2:**
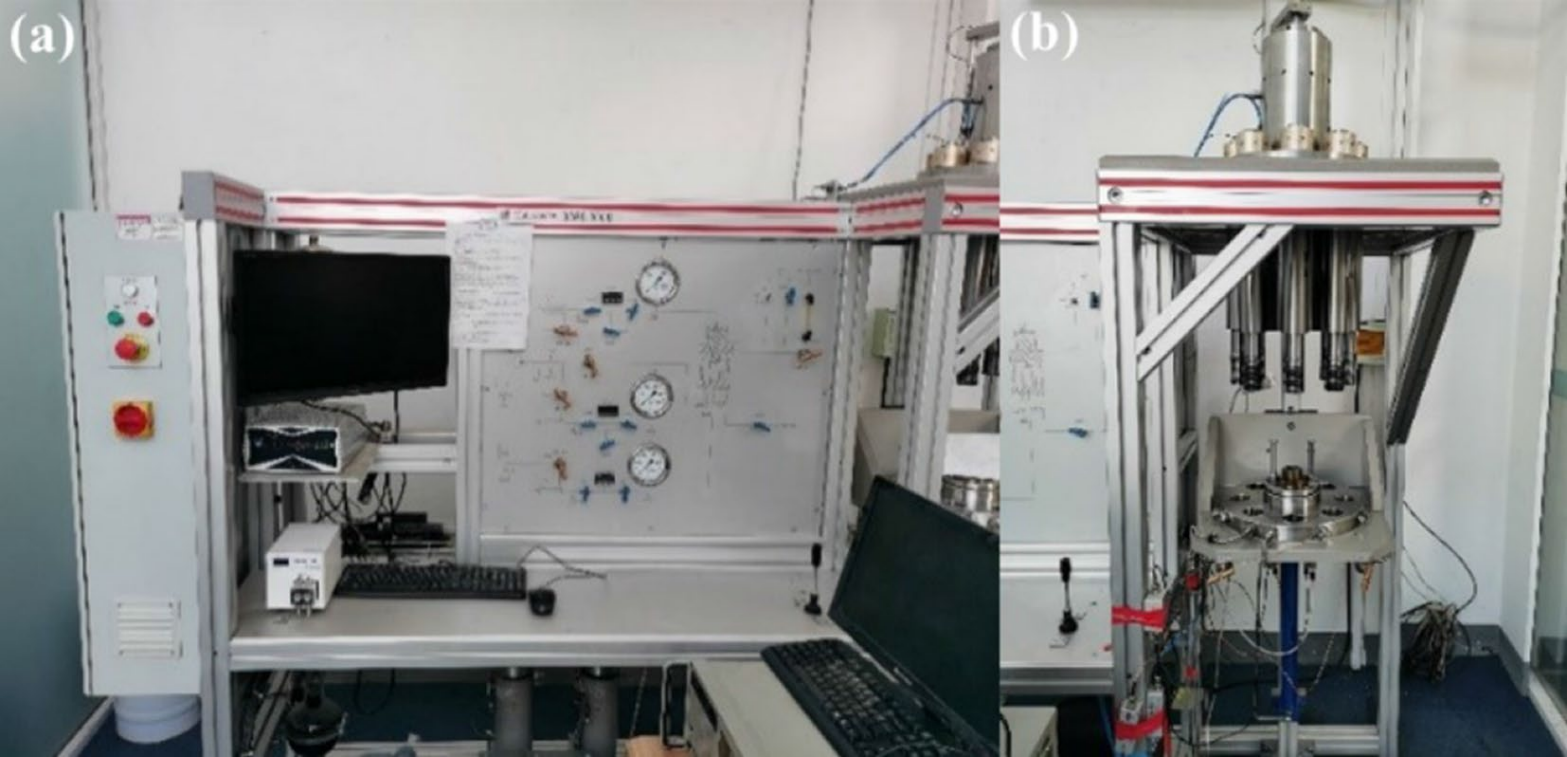
Stac 600–600 rock triaxial rheological test system. (**a**) Data acquisition system. (**b**) Loading system.

Triaxial compression test was carried out on specimens after different freeze–thaw cycles. As the surrounding rock at the tunnel entrance is prone to freeze–thaw damage, and the confining pressure of this part is low. Therefore, 2 MPa confining pressure is selected for test according to actual conditions of tunnel engineering^13^. Stress-controlled loading was used for the test with a constant stress rate of 0.05 MPa/s. The sandstone triaxial compression test uses STAC 600–600 rock triaxial rheological test system. It is suitable for triaxial mechanical test of geological materials such as rock and concrete, and mainly consists of loading system and data acquisition system, as shown in Fig. 2.

The AW21C acoustic emission detector was switched on synchronously during the whole process of triaxial compression test of the specimen. This instrument is used to detect the AE signal. The test parameters of the acoustic emission detector were set as follows: sampling frequency was 10 MHz, gain was 30 dB, threshold value was 35 dB, impact definition time was 50 μs, impact interval time was 300 μs, adjusting threshold voltage was 1.0 V.

### Measuring index

#### Triaxial compression test

Conventional mechanical parameters (peak strain, TCS, modulus of elasticity, Poisson ratio) of each group of sandstone were obtained by triaxial compression test. Therefore, the influence of freeze–thaw cycles on the mechanical properties of sandstone were analyzed.

#### Pore size distribution based on MIP

The deterioration mechanism of rock under freeze–thaw is very complex, but the change of rock microstructure and composition is the main factor affecting the regular change of its physical and mechanical properties. The variation rule of pore size distribution of sandstone can be quantitatively analyzed using MIP^30,31^. Pores are classified into four categories by the boundaries of 1 μm, 10 μm and 100 μm, i.e. small pore (< 1 μm), medium pore (1–10 μm), large pore (10–100 μm) and super-large pore (> 100 μm). The classification of rock pore is mainly based on the size and function of pore.

#### AE signal

AE can detect the process of micro-crack initiation, propagation and formation of macro-crack in rock under load. AE ringing count refers to the number of times that the ringing pulse exceeds the threshold signal in a unit time. By analyzing this parameter, the change of internal structure of sandstone under pressure can be obtained. This paper mainly studied the acoustic emission ringing number and cumulative ringing number produced by sandstone during compression process after different freeze–thaw cycles, which can reflect the difference of freeze–thaw damage in sandstone under different freeze–thaw cycles.

## Analysis of physical properties of sandstone

**Figure 3:**
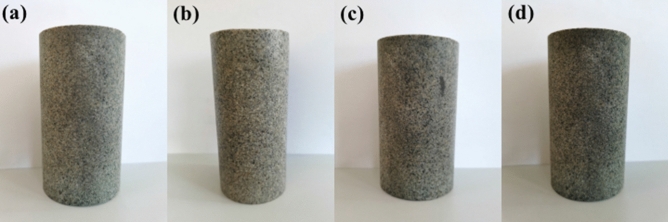
Apparent characteristics of sandstone after different freeze–thaw cycles. (**a**) 0 freeze–thaw cycles. (**b**) 7 freeze–thaw cycles. (**c**) 14 freeze–thaw cycles. (**d**) 21 freeze–thaw cycles.

**Figure 4:**
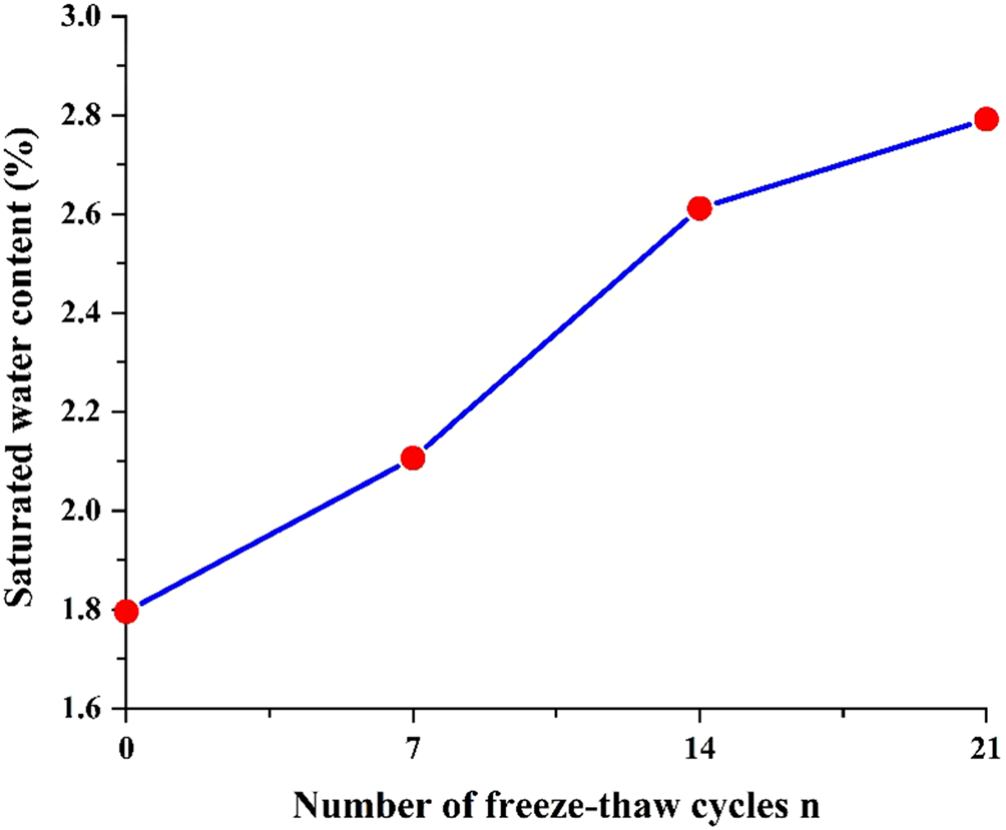
Saturated water content of sandstone with different freeze–thaw cycles.

The saturated water quality of each group of saturated sandstone specimens was measured by test. It was observed that there are no cracks, breakage and spalling of each group of sandstone specimens after freeze–thaw (Fig. 3). Thus, the saturated water content of sandstone after different freeze–thaw times can be estimated. The saturated water content of sandstone after different freeze–thaw cycles is shown in Fig. 4. The average saturated water content was 1.80, 2.08, 2.65, and 2.88% for the freeze–thaw cycles number of 0, 7, 14, 21 times. With the increase of freeze–thaw times, the saturated water content of sandstone shows an overall upward trend, which indirectly indicates that sandstone porosity gradually increases.

## Analysis of mechanical properties of sandstone

### Stress–strain curves

**Figure 5:**
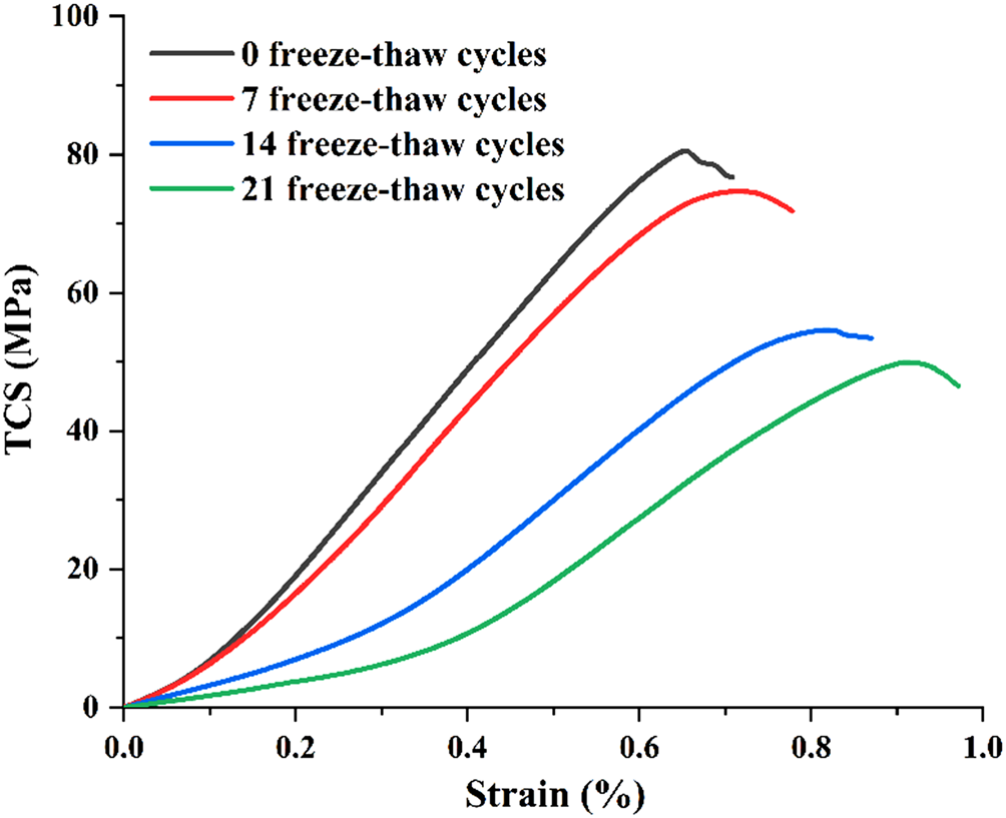
Stress–strain curves.

Triaxial compression tests were carried out on saturated sandstone specimens subjected to different freeze–thaw cycles (0, 7, 14, 21) at room temperature. The stress–strain curves of sandstone under different freeze–thaw times are shown in Fig. 5.

The stress–strain curves obtained from the test specimens after different freeze–thaw cycles have similar shapes and show obvious brittle failure characteristics. During the compression stage of the stress–strain curve of the specimens, defects such as pore and micro crack in the sandstone are compressed and closed. With the increase of the number of freeze–thaw cycles, the compression stage of the stress–strain curve of the specimens prolongs relatively and the concave arc of the curve increases. This phenomenon indicates that the more freeze–thaw cycles occur, the more micro-fissures are present in the sandstone specimens and the greater the overall damage to the sandstone.

The peak strain of samples increases with the increase of freeze–thaw cycles, which is mainly due to the secondary development of natural micro-fissures in sandstone and uneven shrinkage and expansion of mineral particles in sandstone, resulting in the increase of plasticity of sandstone. The peak strain of sandstone after 7 freeze–thaw cycles is 0.07% higher than that of unfreeze–thaw samples. When the number of freeze–thaw cycles exceeds 7 times, the peak strain of the specimens with repeated freeze–thaw cycles changes significantly, increasing by 0.10% and 0.09% respectively in the intervals of 7 to 14 and 14 to 21 times.

### TCS

**Figure 6:**
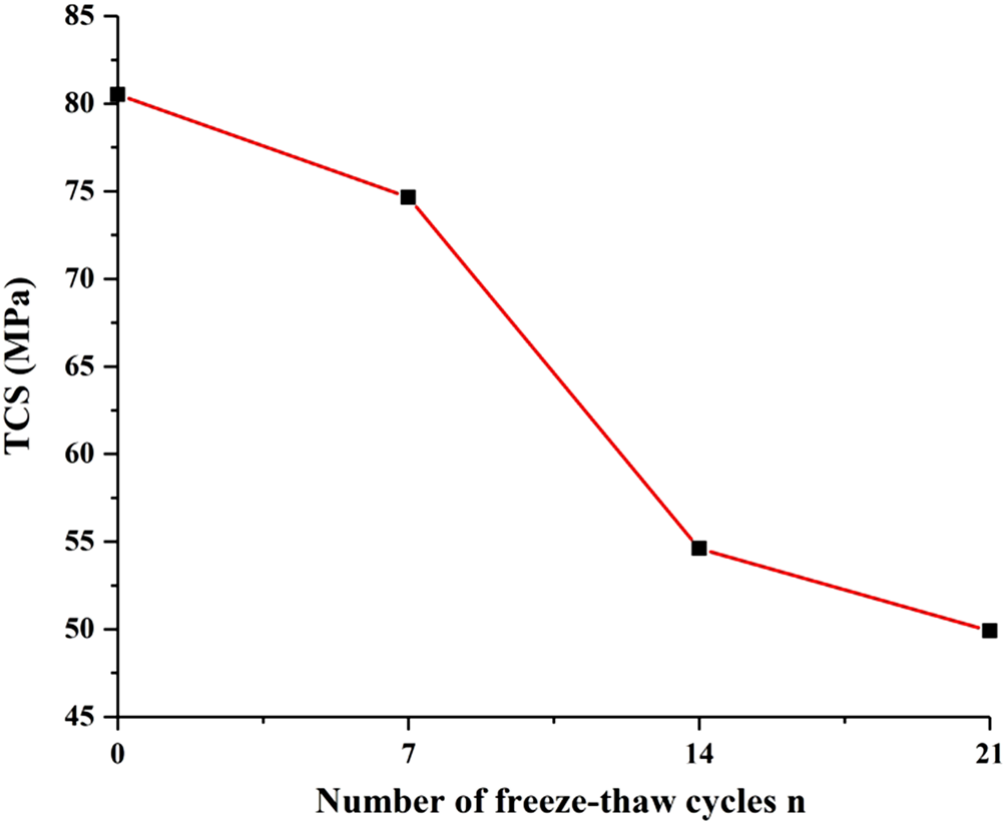
TCS of sandstone with different freeze–thaw cycles.

The TCS of sandstone under different freeze–thaw cycles is shown in Fig. 6. The TCS of saturated sandstone samples decreased with the increase of freeze–thaw times. TCS of sandstone was 80.52, 74.66, 54.61, and 49.96 MPa for the freeze–thaw cycles number of 0, 7, 14, 21 times.

The decreasing rate of sandstone TCS was not stable. The strength of sandstone decreases by 20.05 MPa during the 7th to 14th freeze–thaw cycles. In practical engineering, it is easy to lose structural stability due to the excessive speed of intensity decay in this stage, so more precautions are needed.

### Elastic modulus

**Figure 7:**
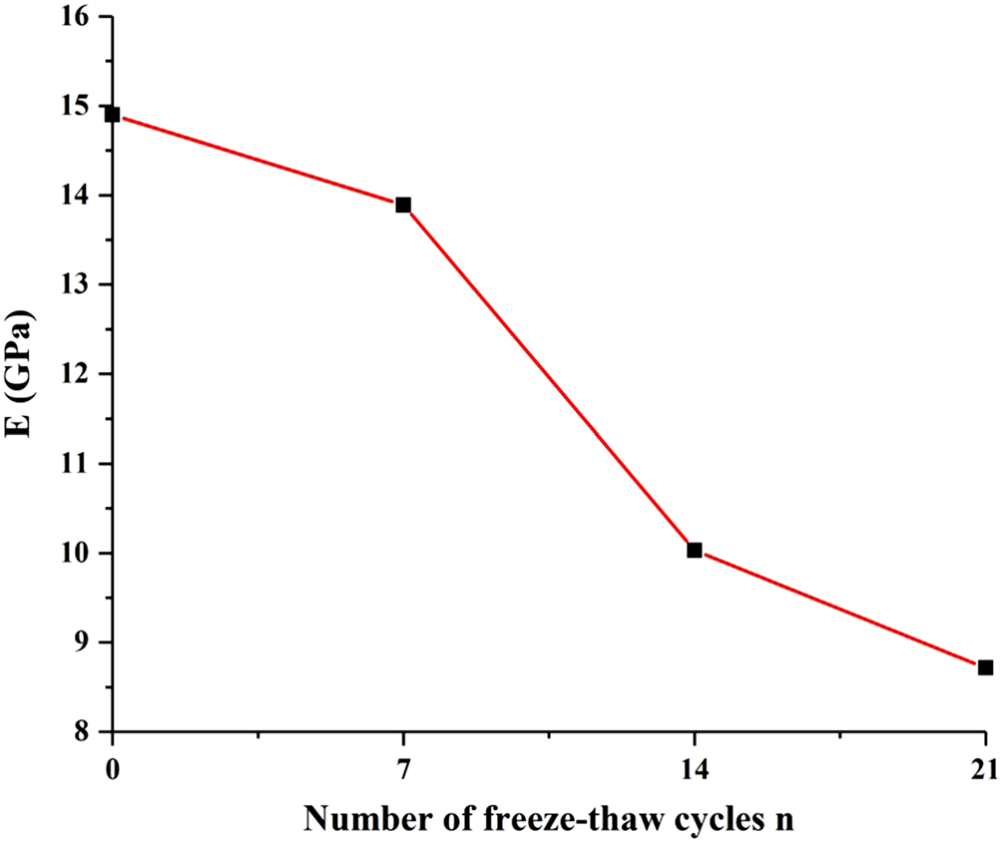
Elastic modulus of sandstone with different freeze–thaw cycles.

The change of the average elastic modulus of sandstone with different freeze–thaw cycles is shown in Fig. 7. The elastic modulus of sandstone decreased gradually with the increase of freeze–thaw times. Elastic modulus of sandstone was 14.90, 13.95, 9.96, and 8.81 GPa for the freeze–thaw cycles number of 0, 7, 14, 21 times. The elastic modulus of sandstone decreased by 4 GPa during the 7th to 14th freeze–thaw cycles, which indicated that sandstone suffered the most severe freeze–thaw damage during the process.

### Poisson ratio

**Figure 8:**
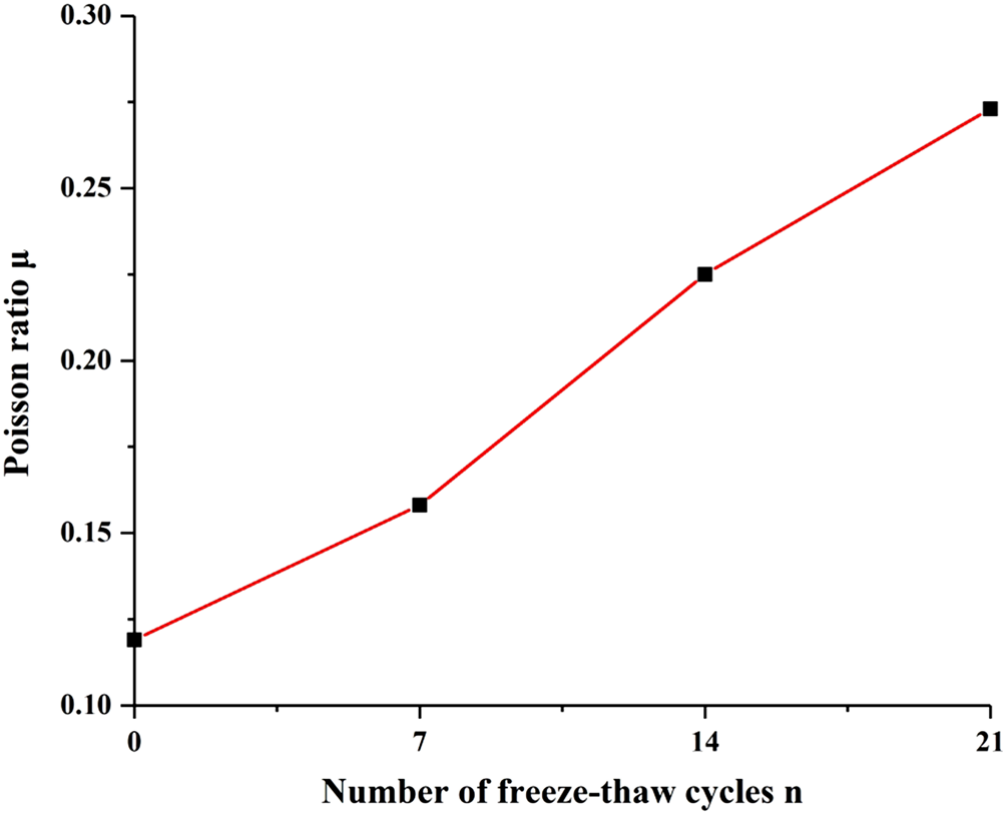
Poisson ratio of sandstone with different freeze–thaw cycles.

Poisson ratio of sandstone under different freeze–thaw cycles is shown in Fig. 8. Poisson ratio of sandstone gradually increases with increasing freeze–thaw times. Poisson ratio of sandstone was 0.12, 0.16, 0.22, and 0.27 for the freeze–thaw cycles number of 0, 7, 14, 21 times. Thus, the Poisson ratio of sandstone can be approximated as a linear increase with the increase of freeze–thaw times.

### Comprehensive analysis

The sandstone texture taken in this test is relatively loose and its porosity is relatively high. After many freeze–thaw cycles, the TCS and elastic modulus of sandstone have decreased, and the peak strain and Poisson ratio have been increased, with obvious change rules. The above proves that freeze–thaw damage has outstanding effect on sandstone in this area. Thus, it is of great engineering significance to determine the mechanical properties of rock after freeze–thaw for calculating the service life and stability of geotechnical engineering in cold area.

In order to clarify the corresponding relationship between freeze–thaw times and mechanical parameters of sandstone samples, the degree of change of sandstone TCS, elastic modulus and Poisson ratio under the action of freeze–thaw in different stages is quantitatively analyzed. The obtained results can predict the mechanical characteristics of sandstone in this geotechnical engineering after different times of seasonal alternation and can be used to guide the construction and maintenance of the engineering.

## Analysis of sandstone damage characteristics

### Macro-destruction form of sandstone

**Figure 9:**
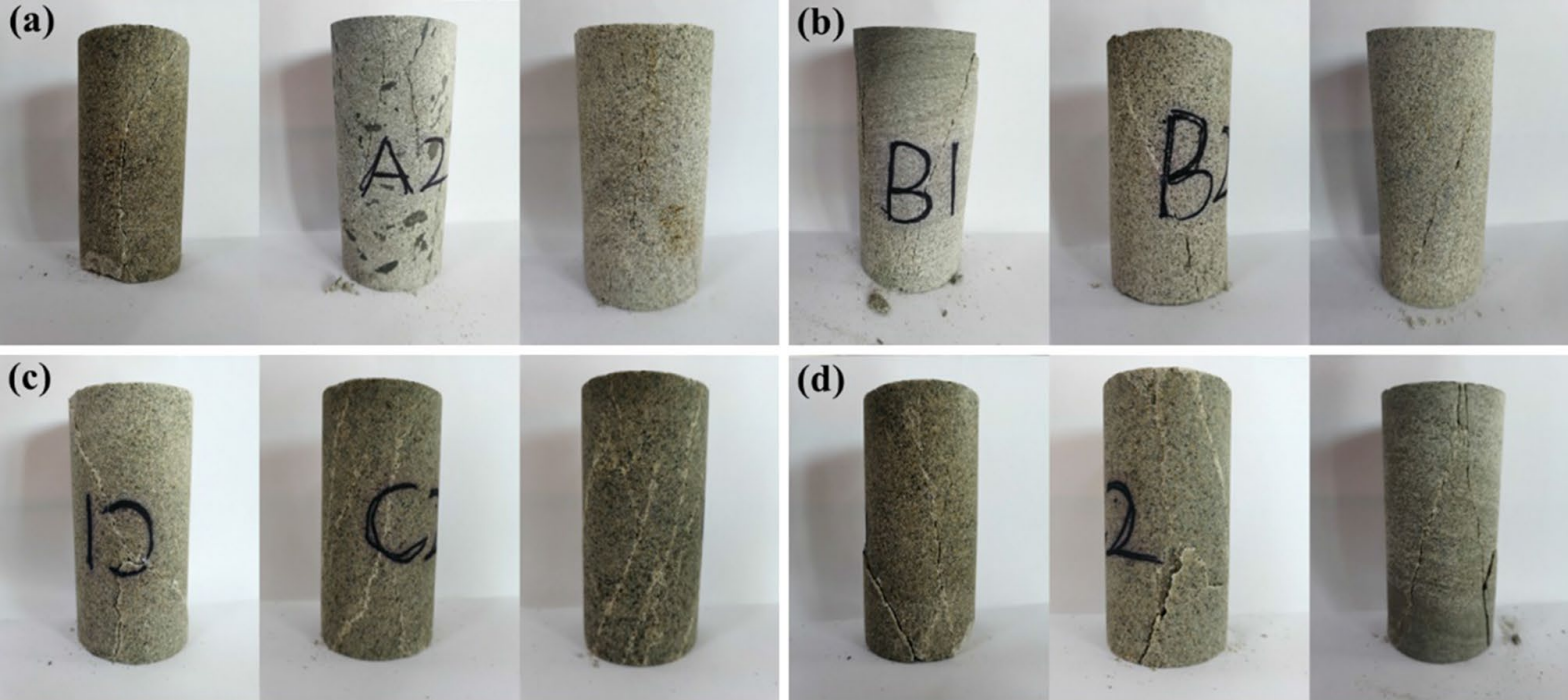
Triaxial compression failure mode of sandstone. (**a**) 0 freeze–thaw cycles. (**b**) 7 freeze–thaw cycles. (**c**) 14 freeze–thaw cycles. (**d**) 21 freeze–thaw cycles.

After freeze–thaw cycles of different times, the failure modes of sandstone after triaxial compression test differ significantly from each other (Fig. 9).

Sandstone that had not undergone freeze–thaw shows splitting failure after triaxial compression, while sandstone that had been frozen-thawed for seven times shows monoclinic shear failure. Compared with the specimens which have experienced less freeze–thaw cycles, the specimens which have experienced more freeze–thaw cycles have a greater extent of fracture propagation. With the increase of freeze–thaw times, the sandstone fracture surface becomes rough gradually, and the secondary fracture surface gradually occurs around the main fracture surface and rock particles fall off. The degree of sandstone fracture continues to increase after 21 freeze–thaw cycles, even bringing about fragments at the end of the specimen during the destruction process. The main reasons for rough and uneven failure surface are that freeze–thaw action weakens the cohesion between sandstone particles, loose internal structure of sandstone and weak shear resistance under pressure.

### Micro-characteristics of sandstone based on MIP

The pore size analysis of sandstone under different freeze–thaw cycles is shown in Table 2. With the increase of freeze–thaw cycles, the proportion of super-large pore and large pore in sandstone increased, while the proportion of medium pore and small pore decreased. When the number of freeze–thaw cycles is 0, 7, 14 and 21, the proportion of super-large pore and large pore in sandstone was 12.44, 14.87, 22.32 and 22.54% respectively, while the proportion of medium pore and small pore was 87.36, 85.13, 77.68 and 77.46% respectively. This indicated that with the increase of freeze–thaw times, the medium and small pore in sandstone gradually develop into super-large and large pore, and the porosity of sandstone increases. The test results prove that the change of micro pore structure of sandstone under freeze–thaw cycle is an important factor affecting its macro-physical and mechanical properties.

**Table 2:** Pore size distribution of sandstone under different freeze–thaw cycles (%).

Pore type	Freeze–thaw cycles number			
	0	7	14	21
small pore	70.28	67.83	62.67	63.97
medium pore	17.28	17.30	15.01	13.49
large pore	8.62	10.72	12.40	9.15
super-large pore	3.82	4.15	9.92	13.39

The percentage of super-large pore increased from 4.15% to 9.92% during 7th-14th freeze–thaw process. This is consistent with the analysis results of saturated water content and mechanical properties of sandstone: Freeze–thaw damage mainly occurs during 7 to 14 freeze–thaw cycles. After more than 14 freeze–thaw cycles, the influence of freeze–thaw on this sandstone specimen is reduced.

### AE characteristics of sandstone

**Figure 10:**
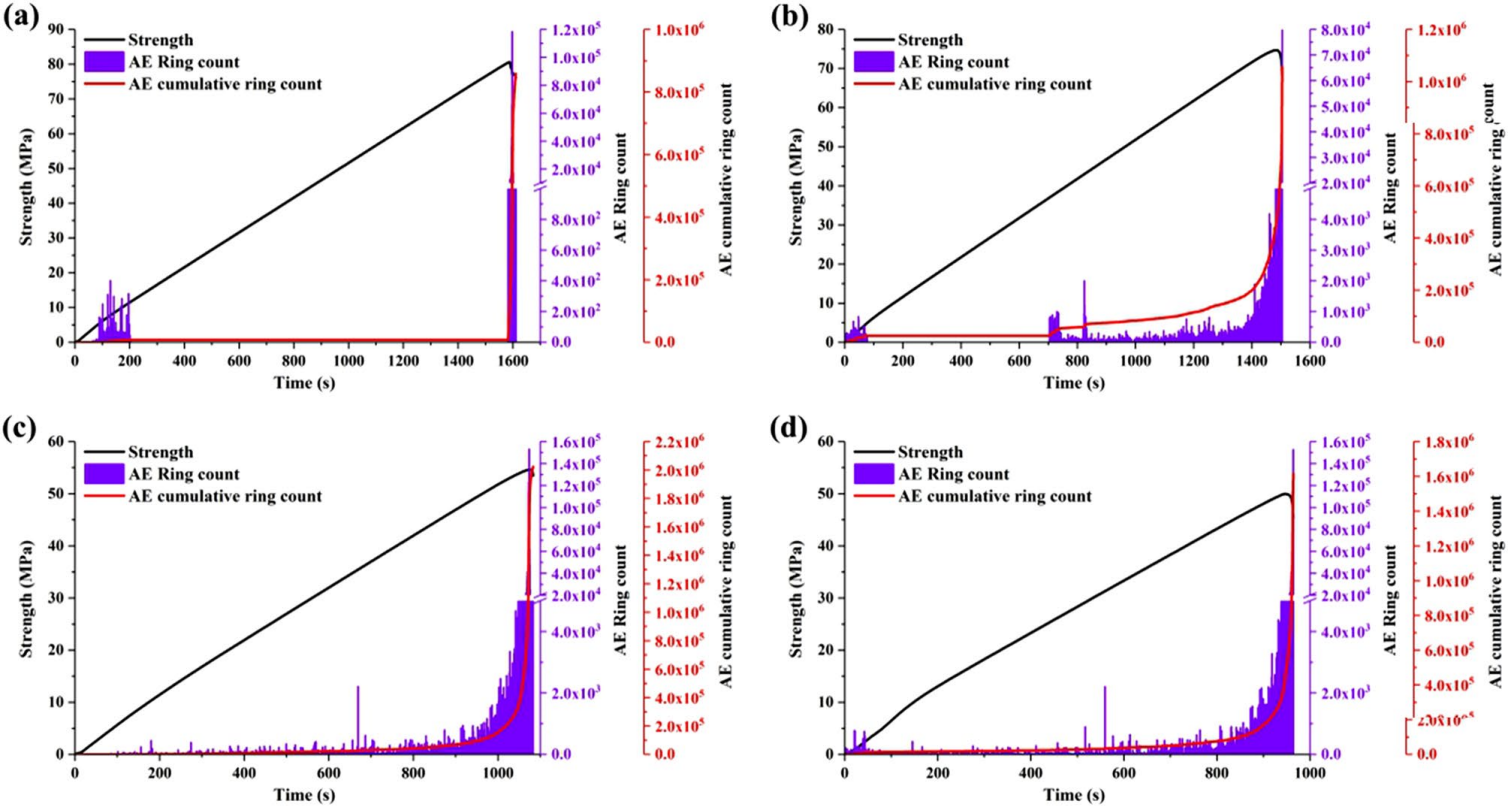
AE ring counts of sandstone under different freeze–thaw times (**a**) 0 freeze–thaw cycles. (**b**) 7 freeze–thaw cycles. (**c**) 14 freeze–thaw cycles. (**d**) 21 freeze–thaw cycles.

The variation law of ringing count and accumulated ringing count of sandstone with time is shown in Fig. 10.

From the diagram that sandstone generates a part of AE signal at the initial stage of loading. This is due to the closure of micro-fissures in sandstone under load and the release of potential energy stored inside the sandstone sample resulting in elastic waves. The AE ring counts of sandstone were sparse in the elastic stage, because no substantial damage has occurred to the sandstone during the elastic stage. The AE ring counts are mainly produced by friction and compaction between rock particles. During the destruction period, the AE ringing counts of sandstone increase rapidly and reach obvious peak value, which indicates that cracks develop rapidly in sandstone until macroscopic damage occurs and a large amount of potential energy stored in sandstone is released. At the same time, the sandstone is completely destabilized and loses its bearing capacity.

As the number of freeze–thaw cycles increases, the damage inside the sample increases, and the micro-cracks gradually increase and merge with each other. The AE signals generated during the destruction process of the specimens gradually become active and the AE cumulative ring counts of gradually increase. The image shows that the AE signal test results of 14 times of freeze–thaw sandstone and 21 times of freeze–thaw sandstone have strong similarity. This phenomenon is consistent with the damage regularity of microstructures of samples obtained based on MIP.

## Conclusion

In this study, different freeze–thaw cycles were carried out on sandstone specimens from an in-service tunnel in northern China. Four groups of physical–mechanical parameters and internal structure damage characteristics of sandstone after different freeze–thaw weathering erosion times were obtained by mechanical test, MIP and AE signal, and some rules were obtained. The test results are analyzed together with the actual engineering conditions. The results of this study can be summarized as follows:

1. With the increase of freeze–thaw times, the saturated water content of sandstone increases by 15.56–60.00%, peak strain and Poisson ratio increase, TCS and elastic modulus decrease by 7.28–37.96% and 6.38–40.87% respectively. After several freeze–thaw cycles, the permeability of sandstone increases and its stability decreases.
2. The failure form of specimen after freeze–thaw changes from splitting destruction to shear failure. With the increase of freeze–thaw cycles, the main fracture surface under pressure becomes rough and shear failure surface rock particles fall off gradually, and the pore distribution of sandstone changes, and the proportion of extra-large and large pore increases from 12.44% to 22.54%.
3. The images of the AE ring counts of sandstone with different freeze–thaw cycles are basically similar. With the increase of freeze–thaw times, the AE signal density of the sample in each stage increases, the AE ring counts appears multiple peaks, and the AE cumulative ring count generally shows an upward trend.

## Data Availability

The data used to support the findings of this study are included within the article.
